# Link between plasminogen activator inhibitor-1 and cardiovascular risk in chronic hepatitis C after viral clearance

**DOI:** 10.1038/srep42503

**Published:** 2017-02-13

**Authors:** Ming-Ling Chang, Yu-sheng Lin, Li-Heng Pao, Hsin-Chih Huang, Cheng-Tang Chiu

**Affiliations:** 1 Liver Research Center, Division of Hepatology, Department of Gastroenterology and Hepatology, Chang Gung Memorial Hospital, Taoyuan, Taiwan; 2 Department of Medicine, College of Medicine, Chang Gung University, Taoyuan, Taiwan; 3 Department of Cardiology, Chang Gung Memorial Hospital, Taoyuan, Taiwan; 4 Healthcare center, Chang Gung Memorial Hospital, Taoyuan, Taiwan; 5 Graduate Institute of Health-Industry Technology, Chang Gung University of Science and Technology, Taoyuan, Taiwan; 6 Research Center for Industry of Human Ecology, Chang Gung University of Science and Technology, Taoyuan, Taiwan.

## Abstract

The pathophysiological implications of plasminogen activator inhibitor-1 (PAI-1) in HCV infection remain obscure. This prospective study evaluated 669 HCV patients, of whom 536 had completed a course of anti-HCV therapy and had pre-, peri- and post-therapy measurements of various profiles, including PAI-1 levels. Multivariate analysis demonstrated, before anti-HCV-therapy, platelet count and PAI-1-rs1799889 genotype were associated with PAI-1 levels. Among patients with a sustained virological response (SVR, n = 445), platelet count was associated with PAI-1 level at 24 weeks post-therapy. GEE analysis showed that PAI-1-rs-1799889 and interferon-λ3-rs12979860 genotypes affected PAI-1 levels early and late in therapy, respectively. At 24 weeks post-therapy, higher lipid, brain natriuretic peptide, homocysteine and PAI-1 levels and PAI-1 activity were noted only in SVR patients compared with pre-therapy levels. Within 24 weeks post-therapy, 2.2% of the SVR (mean age: 57.8 yr; 8 smoking males; the 2 females had pre-therapy hypercholesteremia or cardiovascular family history of disease) and 0% of the non-SVR patients experienced a new cardiovascular event. Platelet counts consistently correlated with PAI-1 levels regardless of HCV infection. PAI-1-rs-1799889 and interferon-λ3-rs12979860 genotypes mainly affected PAI-1 levels longitudinally. Within 24 weeks post-anti-HCV therapy, the SVR patients showed increasing PAI-1 levels with accelerating cardiovascular risk, especially the vulnerable cases.

Hepatitis C virus (HCV), a human pathogen responsible for acute and chronic liver disease, has variants that are classified into 7 major genotypes and has infected an estimated 170 million individuals worldwide^1^. HCV infection is believed to cause metabolic alterations, including steatosis, hypolipidemia, insulin resistance (IR), diabetes and obesity^2^. Despite the favorable lipid profile that results from HCV infection, many studies have shown that HCV infection unfavorably impacts cardiovascular events after adjusting for conventional risk factors^2^,^3^. These results suggest that HCV might affect cardiovascular events via pathways other than virus-induced metabolic modifications, such as IR and hypolipidemia, which may balance each other^2^,^3^. Although most HCV infections are curable using potent direct-acting anti-viral agents, not all HCV-associated cardiometabolic complications are reversible after viral clearance^2^. Free fatty acids and glycerol derived from visceral adipose tissue travel to the liver and stimulate lipoprotein synthesis and gluconeogenesis, respectively. Moreover, adipose tissue exerts important endocrine and immune functions through adipokines^1^,^4^. Thus, dissecting the relationship between HCV infection and adipokine alterations holds promise for preventing or treating HCV-associated cardiovascular complications.

Among the adipokines, plasminogen activator inhibitor-1 (PAI-1), a single-chain glycoprotein with a molecular weight of 50 kDa that acts as a serine protease inhibitor (serpin)^4^, is a principal inhibitor of fibrinolysis due to its inactivation of plasminogen activators. PAI-1 is synthesized and secreted by ectopic fat depots, endothelial cells, hepatocytes, tumor cells and inflammation-activated cells and is present as a stored product in platelets^4^,^5^. The secretion of PAI-1 is stimulated by insulin, free fatty acids, atherogenic lipoproteins and chronic inflammation^6^. Consequently, PAI-1 levels are elevated during thrombotic, fibrotic, and cardiovascular events, as well as in the presence of hyperinsulinemia, hyperlipidemia, hypertension, non-alcoholic fatty liver disease and malignancy, especially those cancers with metastatic behavior and poor prognosis^4^,^5^,^6^,^7^. In contrast, PAI-1 secretion decreases with weight loss^8^. Additionally, PAI-1 works as an early response protein to modulate hepatocyte growth and differentiation^9^ and as a marker of senescence^10^. Previous studies have suggested that a single guanosine insertion/deletion (4 G/5 G) polymorphism in the promoter region and another nearby single nucleotide polymorphism (SNP) in the PAI-1 gene [SERPINE1 (7q22.1)], as well as several SNPs in chromosomes other than chromosome 7, are associated with circulating levels of PAI-1^11^. These polymorphisms potentially increase the risk of IR and thrombosis formation^12^,^13^. With regard to the 4 G/5 G polymorphism, the 4 G allele is associated with moderately high PAI-1 levels. Furthermore, possible gene–environment interactions complicate this association, as differences in PAI-1 levels between individuals with the 4 G vs. the 5 G allele are more apparent in the presence of diseases that stimulate PAI-1 expression^14^. The dynamics of PAI-1 are particularly unpredictable in patients with chronic hepatitis C (CHC), as hyperfibrinolysis may occur due to elevated tissue-type plasminogen activator (tPA) levels^5^. In addition, HCV genotype-specific sustained virological response (SVR)-associated lipid alterations^2^,^15^ might complicate the transcriptional regulation of PAI-1^1^,^2^. Given its complex gene/environmental coordination, the role of PAI-1 in cardiovascular risk for CHC patients remains elusive. Therefore, we sought to address the aforementioned enigmas by conducting a prospective study of PAI-1 levels in patients infected with various genotypes of HCV who had completed anti-HCV therapy. The study was adjusted for crucial confounders, including demographic characteristics as well as metabolic, liver, viral and genetic profiles.

## Results

### PAI-1-rs1799889 genotype correlated with pre-therapy PAI-1 levels in patients with CHC

The baseline (pre-therapy) characteristics of the patients with CHC are listed in Table 1. Patients with an SVR had lower levels of HCV RNA, homeostatic model assessment of insulin resistance (HOMA-IR) and brain natriuretic peptide (BNP) and a lower prevalence of cirrhosis and genotype 1 (G1) HCV infection. However, they had higher prevalences of G2 HCV infection and the interferon-λ3 (IFNL3) CC genotype and higher platelet counts than the non-SVR patients. No notable differences were found in PAI-1 levels between the patients with and without an SVR.

Among the 5 PAI-1-associated SNPs evaluated in the patients with CHC, correlation tests showed that only the 4 G/5 G polymorphism of PAI-1-rs1799889 correlated with pre-therapy PAI-1 levels (Pearson’s correlation coefficient = 0.124, *p* = 0.044).

### Independent factors associated with pre-therapy PAI-1 levels in patients with CHC

Before anti-HCV therapy, as listed in Table 2, univariate and multivariate regression analyses showed that age, body mass index (BMI), platelet count and triglyceride (TG) levels as well as the PAI-1-rs1799889 4 G/4 G genotype were associated with PAI-1 levels. HCV genotype and white blood cell (WBC) count were associated with HCV RNA expression. HCV genotype, BMI, liver cirrhosis and IFNL3 genotype were associated with SVR.

### Independent factors associated with the longitudinal trend in PAI-1 levels in patients with CHC

**Figure 1:**
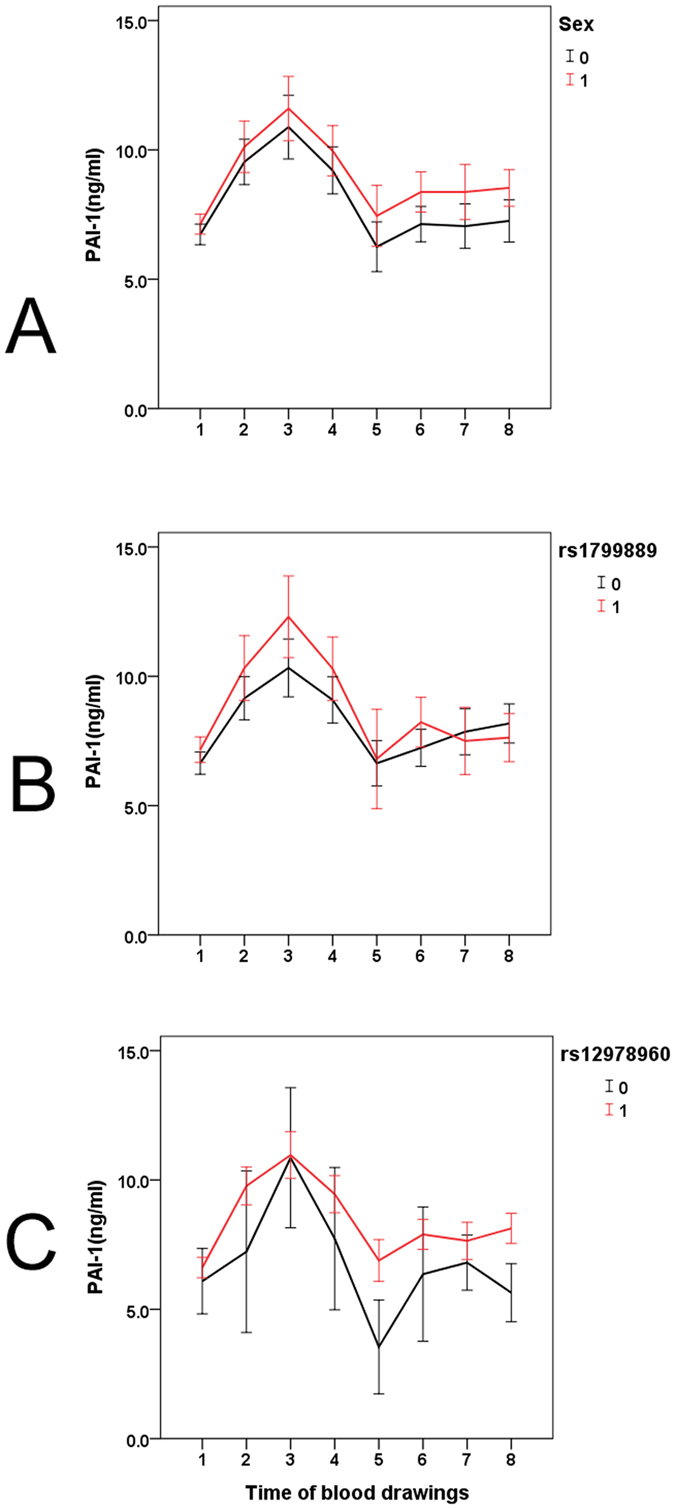
Longitudinal trends in plasminogen activator inhibitor-1 (PAI-1) levels. The included patients with chronic hepatitis C were stratified according to (**A**) sex (male: 1; female: 0), (**B**) PAI-1-rs-1799889 genotype (4 G/4 G genotype: 1; non-4G/4 G genotype: 0) and (**C**) IFNL3-rs-12979860 genotype (CC genotype: 1; non-CC genotype: 0). The blood-drawing time points were as follows: 1, 2 weeks before therapy; 2, after 4 weeks of therapy; 3, after 12 weeks of therapy; 4, after 24 weeks of therapy; 5, after 36 weeks of therapy; 6, after 48 weeks of therapy; 7, after 60 weeks of therapy; and 8, after 72 weeks of therapy.

Using the GEE method, the factors longitudinally affecting PAI-1 levels were identified and are listed in Table 3. We found that sex, BMI, treatment, HOMA-IR, aspartate aminotransferase (AST) to platelet ratio index (APRI), platelet count, estimated glomerular filtration rate (eGFR), total cholesterol (TC), TG levels and PAI-1-rs1799889 and IFNL3-rs12979860 genotypes were independently associated with PAI-1 levels. Among these factors, the impacts of the categorical variables sex and PAI-1-rs1799889 and IFNL3-rs12979860 genotypes on PAI-1 levels were further elucidated and are presented in Fig. 1. The following observations were made: (1) Male patients consistently had higher PAI-1 levels than female patients throughout the therapy (Fig. 1A). (2) The positive impact of the PAI-1-rs 1799889 4 G/4 G genotype on PAI-1 level was most evident at the beginning of the therapeutic course and diminished as the course proceeded (Fig. 1B). (3) Patients with the IFNL3-rs12979860 CC genotype had higher PAI-1 levels than those with the non-CC genotype in the later part of the therapeutic course compared to the beginning (Fig. 1C).

### Independent factors associated with post-therapy PAI-1 levels among patients with an SVR

Among the patients who achieved an SVR, univariate analysis and multivariate analysis confirmed that sex (estimated β = 0.934, *p* = 0.031), age (estimated β = -0.074, *p* = 0.001), platelet count (estimated β = 0.026, *p* < 0.001) and HOMA-IR levels (estimated β = 0.214, *p* = 0.001) were independent factors associated with PAI-1 levels at 24 weeks post-therapy (Supplementary Table 1).

### Only patients with an SVR had increased PAI-1, BNP and homocysteine levels 24 weeks post-therapy

As shown in Table 4, paired *t*-tests demonstrated that the patients with an SVR had decreased ALT and APRI levels as well as increased TC, TG, PAI-1, BNP and homocysteine levels at 24 weeks post-therapy compared with pre-therapy levels. In contrast, the patients with no SVR did not exhibit these changes. Moreover, although the patients with an SVR had lower pre-therapy BNP levels than those without (Table 1), by 24 weeks post-therapy, the difference vanished (*p* = 0.382).

### Increased PAI-1 activity but decreased tPA-PAI-1 complex and tPA levels were observed in patients with an SVR at 24 weeks post-therapy

Among the patients with an SVR, post-therapy PAI-1 activity was elevated (16.7+/−8.6 vs. 10.45+/−9.2 U/ml, *p* = 0.007), while tPA (5.5+/−2.9 vs. 8.3+/−2.3 ng/ml, *p < *0.001) and tPA-PAI-1 complex (840.6+/−712.5 vs. 1470+/−829.7, *p < *0.001) levels were decreased compared with pre-therapy levels. No significant difference was observed between pre- and post-therapy tPA activity (0.017+/−0.23 vs. 0.029+/−0.048 IU/ml, *p* = 0.09).

**Figure 2:**
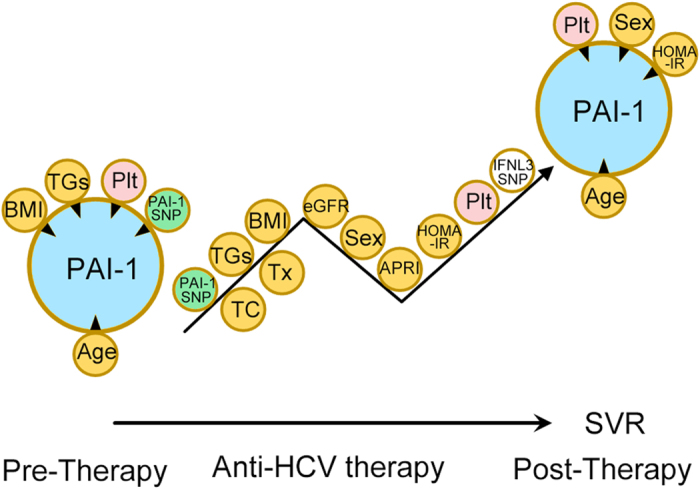
Associations between independent factors and plasminogen activator inhibitor-1 (PAI-1) levels in pre-, peri- and post-anti-hepatitis C virus therapy stages. The tips of the black arrowheads indicate dependent factors, and the bases of the black arrowheads indicate independent factors. BMI: body mass index; TGs: triglycerides; Plt: platelet; PAI-1 SNP: PAI-1-rs-1799889 genotype; TC: total cholesterol; Tx: anti-HCV therapy; eGFR: estimated glomerular filtration rate; Sex: male sex; APRI: aspartate aminotransferase to platelet ratio index; HOMA-IR: homeostasis model assessment-estimated insulin resistance; IFNL3: interferon λ3; TC: total cholesterol.

The associations identified between independent factors and PAI-1 levels are shown in Fig. 2.

### New cardiovascular events were noted only in patients with an SVR within 24 weeks post-therapy

Before anti-HCV therapy, metabolic syndrome was noted in 29.1% of the patients. At 24 weeks after anti-HCV therapy, 27% and 0% of the patients with no SVR had metabolic syndrome and cardiovascular events, respectively. In contrast, metabolic syndrome and new advent of cardiovascular events were noted in 27.8% and 2.2% (n = 10) of the patients with an SVR, respectively [cardiovascular events, non-SVR patients (0%) vs. SVR (2.2%), *p* = 0.025]. Among the 10 patients with an SVR who had cardiovascular events, eight were males who smoked. Of those, 3 suffered from acute myocardial infarction (two were rescued by angioplasty with stent implantation, and one was saved by coronary artery bypass grafting), 5 had acute cerebrovascular events (two were cerebral infarctions, one was an intracerebral hemorrhage, and two were transient ischemic attacks). Of the affected female patients, one had a strong family history of cardiovascular events and suffered from acute cerebral infarction. The other had pre-therapy hypercholesteremia and suffered from acute intestinal ischemia due to mesenteric vascular thrombosis. The mean and median age of the 10 patients were 57.8 and 55.0 years old, respectively. Most of the new cardiovascular events occurred 3–6 months after completing the anti-HCV therapy. Of the conventional risks for cardiovascular events, including sex, age, BMI, history of diabetes mellitus, hypertension, dyslipidemia, and smoking^16^, smoking [odds ratio (OR): 0.617, 95% confidence interval (CI) of OR:1.436–26.53] and dyslipidemia (OR: 5.25, 95% CI of OR: 1.27–21.68) were the independent risk factors associated with the advent of new cardiovascular events. There were no differences in the prevalences of these conventional risk factors between those with and without an SVR (Supplementary Table 2).

### Negligible impacts from fibrosis and steatosis on hepatic PAI-1 expression in patients with CHC

**Figure 3:**
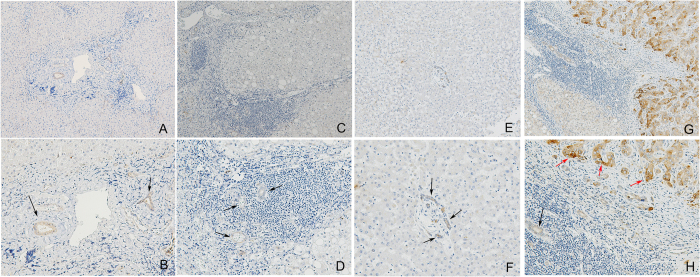
Immunohistochemical studies of hepatic plasminogen activator inhibitor-1 (PAI-1) levels. Representative livers from a chronic hepatitis C (CHC) patient with severe fibrosis and some inflammation (**A**, 100X; **B**, 200X) and another CHC patient with severe inflammation, steatosis and mild fibrosis (**C**, 100X; **D**, 200X) before the initiation of anti-hepatitis C virus therapy. A liver specimen from a normal subject was stained for PAI-1 (**E**, 100X; **F**, 200X) and served as a negative control (without HCV infection). A hepatocellular carcinoma specimen served as a positive control for PAI-1 staining (**G**, 100X; **H**, 200X). The arrows indicate PAI-1-positive stained cells. Black arrows: biliary and endothelial cells; red arrows: malignant hepatocytes.

The impact of hepatic fibrosis and steatosis on hepatic PAI-1 expression in CHC patients before anti-HCV therapy were negligible, as only some biliary cells and endothelial cells (Fig. 3A–D, black arrows), but no inflammatory cells or hepatocytes, expressed PAI-1, and the expression was very weak. Moreover, compared with the normal controls (Fig. 3E,F), the CHC patients did not exhibit differences in hepatic PAI-1 expression (0.15+/−0.07% vs. 0.11+/−0.21%, *p* = 0.326). A hepatocellular carcinoma (HCC) sample showed that many malignant hepatocytes were strongly positive for PAI-1 expression (Fig. 3H, red arrows), while the biliary cells weakly expressed PAI (Fig. 3H, arrows). In contrast, normal hepatocytes in the HCC sample, even those close to areas with inflammation, fibrosis or steatosis, did not express PAI-I (Fig. 3G,H).

## Discussion

To the best of our knowledge, this is the first prospective study to elucidate the relationships that exist among HCV infection, alterations in PAI-1 levels and cardiovascular risk factors. The most compelling results are as follows: (1) Patients with an SVR had similar PAI-1 levels but lower pre-therapy BNP levels than patients with no SVR. (2) Among the 5 tested PAI-1-associated SNPs, only PAI-1-rs1799889 correlated with PAI-1 levels in CHC patients. (3) Regardless of HCV infection, platelet counts were positively associated with PAI-1 levels. PAI-1-rs-1799889 and IFNL3-rs12979860 genotypes particularly affected PAI-1 levels early and late in therapy, respectively. (4) Compared with pre-therapy levels, 24-week post-therapy levels of PAI-1, BNP, homocysteine and lipids were primarily increased in patients with an SVR, regardless of HCV genotype. (5) Within 24 weeks post-therapy, only patients with an SVR experienced a new advent of cardiovascular events.

In the current study, all of the differences between the patients with and without an SVR in the measured pre-therapy variables, including viral load and HOMA-IR levels, have been previously clarified^2^,^15^,^17^,^18^, with the exception of BNP levels, which are well-established positive markers of cardiovascular events^19^,^20^. BNP levels were significantly lower in the patients with an SVR than in those without. This phenomenon may be related to the different viral loads rather than the different HOMA-IR levels between these groups because the negative association between BNP and HOMA-IR levels is blunted at pathological BNP levels^21^. For PAI-1, PAI-1 mRNA-binding protein may confer cAMP-mediated regulation of PAI-1 mRNA stability, allowing binding of PAI-1 mRNA to the HCV internal ribosomal entry site (IRES)^22^. This discovery suggests a potential link between HCV IRES biology and PAI-1 in associated cellular functions. Moreover, PAI-1 levels were reported to be predictive of interferon-based therapeutic responses in a cohort of 190 G1 CHC patients^23^. However, in the current study (669 patients with CHC, mainly G1 and G2), no association was noted between pre-therapy PAI-1 and HCV RNA levels, and no predictive role of PAI-1 level in anti-HCV therapeutic response was found. Although inflammation/fibrosis and hepatic steatosis are believed to increase PAI-1 levels^4^,^5^,^6^,^7^, our immunohistochemical (IHC) studies showed that the influence of hepatic fibrosis and steatosis on hepatic PAI-1 expression of CHC patients was negligible. Moreover, APRI, a positive marker of hepatic fibrosis in CHC^24^, was not associated with serum PAI-1 levels in pre- or post-therapy multivariate analyses. Additionally, no differences in hepatic PAI-1 expression were noted between the normal controls and the CHC patients before anti-HCV therapy. Collectively, the impact of HCV infection on PAI-1 level, if any, is likely indirect and of a non-hepatic origin.

Of note, among the 5 PAI-1-associated SNPs tested, only PAI-1-rs1799889 was associated with PAI-1 levels. The CHC patients who were homozygous for the 4 G allele (4 G/4 G) of PAI-1-rs1799889 presented the highest PAI-1 levels. This is consistent with the finding that the protein encoded by the 4 G allele has higher activity than that encoded by the 5 G allele^25^. Because PAI-1 is considered a strong acute-phase reactant and the PAI-1-rs1799889 genotype has been described as a response polymorphism^14^, the impact of the PAI-1-rs1799889 4 G/4 G genotype was most evident in the pre-therapy PAI-1 levels and during the initial stages of the longitudinal trend for PAI-1. This impact vanished after the development of an SVR, when virus-associated inflammation disappeared. In contrast, platelet counts were consistently positively associated with PAI-1 levels, regardless of HCV infection status. These findings highlight the importance of blood platelets as a main source of serum PAI-1^25^. The impact of IFNL3-rs12979860 (which is highly associated with SVR)^1^,^15^ on PAI-1 levels was evident during the later course of therapy, demonstrating that SVR did affect the longitudinal trend of PAI-1 levels. PAI-1 levels increased after SVR in a platelet-, sex-, age- and HOMA-IR-dependent manner, but all the dependent variables remained unchanged. These findings indicate that sources other than platelets, such as visceral adipose tissue^26^, or transcriptional up-regulation subsequent to increased lipid profile^2^,^6^,^15^ might account for the increased PAI-1 levels observed after SVR. Interestingly, BMI decreased but lipid profiles increased (Table 4) after SVR, showing a reciprocal trend in regulating PAI-1 levels^4^,^5^,^6^,^7^. Increased lipid profiles that counterbalanced the down-regulated effect of decreased BMI on PAI-1 levels might therefore be the main driving force for increased PAI-1 levels in CHC patients after SVR. Although the SVR-associated lipid alterations were different between G1 and G2 HCV infection^15^, no genotype-specific SVR-associated PAI-1 alteration was noted. As PAI-1 is necessary for modulating hepatocyte growth and differentiation^9^, the increased PAI-1 levels after SVR might also be associated with hepatocyte regeneration in response to viral clearance. However, increased PAI-1 levels have been strongly linked to increased cardiovascular risk^27^,^28^. One unit of PAI-1 activity is defined as the quantity of PAI-1 that can neutralize one unit of single-chain tPA in 10 minutes^29^. After SVR, PAI-1 activity increased while levels of tPA-PAI-1 complex and tPA decreased. All these alterations led to a hypercoagulable state. Moreover, BNP and homocysteine levels, which are both highly associated with cardiovascular events^19^,^20^, became elevated after the development of an SVR. In particular, pre-therapy levels of BNP were lower in the patients with an SVR than in those without and were supposed to decrease rather than increase after viral clearance. BNP has a role in cardiac protection arising from its capacity to regulate water and electrolyte levels^19^,^20^. Furthermore, BNP has antifibrotic and cytoprotective properties that inhibit adverse myocardial remodeling by suppressing PAI-1 mRNA and protein expression^19^,^20^. However, this inhibition was not effective in the patients with an SVR, as PAI-1 expression increased after the development of an SVR. Within 24 weeks post-therapy, ten (2.2%) of the patients with an SVR experienced a new-advent cardiovascular event. Most of the patients were old males who smoked or females with risk factors for cardiovascular events. In contrast, none of the patients with no SVR experienced any cardiovascular events. The higher incidence of cardiovascular events in the SVR vs. non-SVR group was confirmed by analyses that adjusted for the associated conventional risks. Paradoxically, several cohort studies have shown that anti-HCV therapy is associated with improved cardiovascular outcomes in CHC patients^2^, and a recent comprehensive meta-analysis also showed that these patients are at increased risk for cardiovascular event-related morbidity and mortality^30^. Although viral clearance eliminates hepatic injury, we propose that host metabolic homeostasis is perturbed, at least within 24 weeks post-therapy with interferon-based therapy. Therefore, special caution may be needed for populations that are vulnerable to cardiovascular events^31^. Conversely, for subjects who are free from conventional cardiovascular risks, the benefit of viral clearance would compensate for the temporary perturbation of host homeostasis in eliminating cardiovascular risk. Finally, because PAI-1 levels are predictive of many diseases in addition to cardiovascular events^7^,^8^, the data from the current study provide a reference for the evaluation of other co-morbidities.

Given that adipose tissue is the major source for adipocytokines including PAI-1^4^, one of the major limitations of the current study was the lack of a pathological study of adipose tissue. Further studies of PAI-1 levels in CHC patients should include comprehensive surveys of adipose tissue pathology with transcriptional assays considering the influence of lipid profiles to confirm our findings and elucidate the associated molecular mechanisms. Furthermore, since only 2.2% of the patients with an SVR developed a new cardiovascular event, the difference in cardiovascular events between the patients with and without an SVR, although statistically significant, requires future studies with larger cohorts that can provide higher confirmatory power than the current study.

In summary, regardless of HCV infection, platelet counts were positively associated with PAI-1 levels. The impact of the PAI-1-rs-1799889 and IFNL3-rs12979860 genotypes on PAI-1 levels was evident in the early and late stages of therapy, respectively. At 24 weeks post-therapy, the patients with an SVR had higher levels of PAI-1, BNP and homocysteine and higher PAI-1 activity compared to pre-therapy measurements. Within 24 weeks post-therapy, only the patients with an SVR experienced new-advent cardiovascular events. Cautious follow-up focusing on cardiovascular events might be required for CHC patients who achieve an SVR following interferon-based anti-HCV therapy, particularly for vulnerable patients, at least within 24 weeks post-therapy.

## Materials and Methods

### Patients

The study group comprised subjects 18 years or older who had CHC, defined as detectable HCV RNA for >24 weeks. Subjects with human immunodeficiency virus infection, hepatitis B infection, hemochromatosis, coronary heart disease or malignancy were excluded, as were recipients of solid organ transplants.

### Methods

A total of 669 CHC patients (over 94% were G1 and G2 infections; G3, G6 or mixed infections accounted for <6%) were consecutively recruited from a tertiary referral center between July 2010 and August 2015. Of the 669 patients, 536 received anti-HCV therapy with weight-based pegylated interferon-α-2b and ribavirin according to a response-guided therapeutic protocol^1^,^15^. HCV RNA levels and genotypes as well as IFNL3-rs12979860 SNPs were assessed as previously described^1^,^15^. Preliminary screening tests of 5 PAI-1-associated SNPs, including rs2227669, rs6976053, rs6486122, rs11128603, and rs1799889^11^. were assessed using TaqMan SNP Genotyping assays (Applied Biosystems, Waltham, Massachusetts, USA) (Supplementary Table 3) among 200 patients randomly selected from the total cohort. For all 669 included patients, several baseline factors were evaluated, including sex, age, BMI, HCV RNA expression, HCV genotype, presence of fatty liver and cirrhosis, rs1799889 status, eGFR and APRI, as well as TC, TG, HOMA-IR [fasting insulin (μU/mL) × fasting glucose (mmol/L)/22.5], high sensitivity C-reactive protein (hsCRP), ALT, PAI-1 (R&D Systems, MN, USA), BNP (Sigma-Aldrich Corp., MO, USA) and homocysteine (Axis-Shield Diagnostics Ltd, Dundee, UK) levels. For the 536 patients who completed anti-HCV therapy, the aforementioned factors were evaluated 2 weeks before therapy; after 4, 12 and 24 weeks of therapy; at the end of therapy; and 12 and 24 weeks after the end of therapy. Pre-therapy and 24-week post-therapy serum PAI-1 and tPA activities and tPA (Molecular Innovations, MI, USA) and tPA-PAI-1 complex (Abcam plc. MS, USA) levels were assessed according to manufacturer protocols. Abdominal ultrasound studies were performed to assess the presence of fatty liver and cirrhosis. SVR was defined as undetectable levels of HCV RNA at 24 weeks after the completion of therapy. Metabolic syndrome and cardiovascular events were surveyed and recorded as described previously^32^. Liver biopsy and IHC studies were performed prior to the initiation of anti-HCV therapy in 20 CHC patients. Twenty paraffinized normal liver samples were acquired from the tissue bank of the hospital to perform IHC studies. The hepatic IHC studies of PAI-1 (R&D Systems) were performed using paraffinized liver samples according to the manufacturer’s protocols. Protein expression intensity was determined using Image J software (http://imagej.nih.gov/ij/, National Institutes of Health, USA).

### Statistics

All statistical analyses were performed using Statistical Package for the Social Sciences (SPSS package version 21, SPSS Inc., Chicago, USA) software. Continuous variables were analyzed using Student’s t-test, whereas categorical variables were analyzed using the chi-squared test or Fisher’s exact test, as appropriate. Univariate and multivariate linear regression models were used to assess relationships between various pre-therapy dependent and independent variables. Generalized estimating equation (GEE) repeated measures tests were applied to determine the longitudinal relationships between the dependent and independent variables. Paired t-tests were used to compare variables prior to and at 24 weeks after anti-HCV therapy within individuals. Statistical significance was defined at the 5% level based on two-tailed tests of the null hypothesis.

### Informed consent

Written informed consent was obtained from each patient. The study protocol conformed to the ethical guidelines of the 1975 Declaration of Helsinki and was approved by the Chang Gung Memorial Hospital institutional review board.

## Additional Information

**How to cite this article**: Chang, M.-L. *et al*. Link between plasminogen activator inhibitor-1 and cardiovascular risk in chronic hepatitis C after viral clearance. *Sci. Rep.* **7**, 42503; doi: 10.1038/srep42503 (2017).

**Publisher's note:** Springer Nature remains neutral with regard to jurisdictional claims in published maps and institutional affiliations.

## Supplementary Material

Supplementary Tables

## Figures and Tables

**Table 1:** Baseline characteristics of all the enrolled chronic hepatitis C patients.

	Total, n = 669 (treated and untreated)	SVR (+), n = 455	SVR (−), n = 91	*p-value*s
Male, n (%) #	351 (55.6)	257 (57.8)	47 (52)	0.394
Age (yr)	55.11+/−12.08	53.04+/−12.93	57.5+/−12.47	0.160
BMI	24.95+/−3.95	24.79+/−3.68	25.84+/−4.28	0.057
HCV RNA (Log_10_ IU/ml)	5.97+/−1.12	2.95+/−6.26	5.68+/−4.96	<0.001*
HCV genotype (G), n (%) #				
G1	368 (55.0)	214 (47.1)	72 (79.1)	<0.001*
G2	263 (39.3)	214 (47.1)	17 (18.7)	<0.001*
G3	15 (2.2)	12 (2.6)	0 (0)	0.386
G6	9 (1.3)	6 (1.3)	2 (2.2)	0.335
G1 + G2	7 (1.0)	6 (1.3)	0 (0)	0.595
G1 + G3	1 (0.1)	1 (0.2)	0 (0)	0.832
Unidentified	6 (0.9)	2 (0.8)	0 (0)	0.574
HOMA-IR	3.23+/−5.35	3.02+/−6.56	5.08+/−8.47	0.043*
Hepatic steatosis, n (%) #	311 (46.5)	218 (48)	40 (43.75)	0.373
Liver cirrhosis, n (%) #	140 (20.9)	100 (22)	40 (44.1)	0.001*
ALT (U/L)	92.09+/−94.59	104.7+/−96.0	85.6+/−80.0	0.116
APRI	1.64+/−2.04	1.479+/−1.92	1.758+/−1.986	0.21
hsCRP (mg/dL)	1.68+/−3.71	1.70+/−3.16	1.59+/−1.97	0.752
WBC count (10^3^/μL)	5.66+/−1.90	5.83+/−1.92	5.59+/−1.66	0.486
Platelets count (10^3^/μL)	176.9+/−64.5	182.2+/−58.5	155.6+/−57.7	0.001*
TC (mg/dL)	171.3+/−34.5	168.05+/−32.08	176.29+/−27.90	0.735
TGs (mg/dL)	104.9+/−50.5	98.53+/−44.58	116.71+/−70.32	0.123
PAI-1 (ng/ml)	6.87+/−3.01	6.93+/−3.47	6.30+/−4.48	0.701
BNP (pg/mL)	424.2+/−67	420.5+/−71.7	512.0+/−47.1	0.017*
Homocysteine (*μ*mol/L)	14.0+/−7.44	14.3+/−7.9	13.7+−0.4.5	0.76
eGFR	89.91+/−36.40	82.75+/−34.89	84.39+/−35.82	0.748
PAI-1-rs1799889, 4 G/4 G, n (%) #	285 (42.6)	190 (41.7)	41 (45.1)	0.984
IFNL3-rs12979860CC, n (%) #	564 (84.3)	392 (88.2)	61 (67)	0.003*

#Chi-squared test; SVR: sustained virological response; BMI: body mass index; G: genotype; Log: logarithmic; *p < 0.05; G: genotype; HOMA-IR: homeostasis model assessment-estimated insulin resistance; ALT: alanine aminotransferase; APRI: aspartate aminotransferase to platelet ratio index; hsCRP: high sensitivity C- reactive protein; WBC: white blood cells; TC: total cholesterol; TGs: triglycerides; PAI-1: plasminogen activator inhibitor-1; BNP: brain natriuretic peptide; eGFR: estimated glomerular filtration rate; IFNL3: interferon-λ3.

**Table 2:** Univariate and multivariate analyses of factors associated with pre-therapy HCV RNA and PAI-1 levels as well as SVR in all enrolled chronic hepatitis C patients.

Variants	HCV RNA (Log_10_ IU/ml)		PAI-1 (ng/ml)		SVR	
	Univariate analysis: 95% CI of estimated β (*p-value*s)	Multivariate analysis: 95% CI of estimated β [estimated β] (*p-value*s)	Univariate analysis: 95% CI of estimated β (*p-value*s)	Multivariate analysis: 95% CI of estimated β [estimated β] (*p-value*s)	Univariate analysis: 95% CI of OR [OR] (*p-*values)	Multivariate analysis: 95% CI of OR [OR] (*p-*values)
Sex (Male)	−0.839~2.037 (0.413)		−0.038~1.019 (0.069)		0.707~1.877 [1.151] (0.571)	
Age	−0.068~0.047 (0.721)		−0.079~−0.034 (<0.001*)	−0.062~−0.002 [−0.032] (0.037*)	0.961~1.004 [0.982] (0.11)	
BMI	−0.233~0.167 (0.746)		0.12~0.225 (<0.001*)	0.075~0.265 [0.17] (<0.001*)	0.877~0.977 [0.935] (0.042*)	0.82~0.992 [0.902](0.033*)
HCV genotype	−0.279~−0.14 (<0.001*)	−0.311~−0.098 [−0.204] (<0.001*)	−0.286~0.223 (0.807)		1.361~3.604 [2.214] (0.001*)	2.356~16.156 [0.617] (<0.001*)
HCV RNA (Log_10_ IU/ml)	NA	NA	−0.004~0.49 (0.053)		0.385~0.708 [0.522] (<0.001*)	0.464~1.307 [0.694] (0.075)
ALT (U/L)	−0.011~0.008 (0.777)		−001~0.005 (0.158)		0.999~1.007 [1.003] (0.11)	
APRI	−0.481~0.318 (0.689)		−0.364~−0.008 (0.002)	−0.152~0.337 [0.093] (0.457)	0.802~1.060 [0.922] (0.252)	
hsCRP (mg/dL)	−0.019~0.028 (0.702)		−0.068~0.19 (0.352)		0.92~1.118 [1.014] (0.774)	
WBC count (10^3^/μL)	0.021~0.11 (0.004*)	0.021~0.135 [0.078] (0.008*)	0.223~0.492 (<0.001*)	−0.094~0.298 [0.102] (0.307)	0.905~1.209 [1.046] (0.542)	
Platelets count (10^3^/μL)	0.000~0.002 (0.212)		0.014~0.022 (<0.001*)	0.009~0.025 [0.017] (<0.001*)	1.003~1.012 [1.008] (0.001*)	0.997~1.013 [1.005] (0.216)
TC (mg/dL)	−0.016~0.031 (0.531)		−0.008~0.008 (0.96)		0.992~1.007 [0.999] (0.843)	
TGs (mg/dL)	0.006~0.032 (0.005*)	−0.003~0.002 [−0.001] (0.607)	0.007~0.17 (<0.001*)	0.002~0.016 [0.009] (0.012*)	0.99~0.999 [0.995] (0.019*)	0.991~1.004 [0.917] (0.46)
HOMA-IR	0.086~0.348 (0.001*)	−0.008~0.035 [0.013] (0.231)	−0.038~0.049 (0.813)		0.909~1.004 [0.955] (0.073)	
Hepatic steatosis	−3.06~0.386 (0.128)		0.401~1.529 (0.001*)	−0.284~1.227 [0.472] (0.22)	0.725~2.048 [1.219] (0.455)	
Liver cirrhosis	−0.342~0.069 (0.192)		−1.728~−0.41 (0.001*)	−0.569~1.269 [0.33] (0.47)	0.227~0.673 [0.391] (0.001*)	0.109~0.684 [0.273] (0.006*)
PAI-1 (ng/ml)	−0.297~0.186 (0.65)		NA	NA	0.919~1.117 [1.013] (0.794)	
BNP (Log_10_ pg/mL)	−0.383~0.264 (0.717)		−0.571~1.052 (0.377)		0.128 ~0.828 [0.326] (0.018*)	0.045~0.818 [0.192] (0.026*)
Homocysteine (*μ*mol/L)	−0.044~0.007 (0.155)		−0.12 ~0.017 (0.14)		0.945~1.080 [1.011] (0.758)	−0.044~0.007 (0.155)
eGFR	−0.001~0.004 (0.235)		−0.005~0.011 (0.427)		0.992~1.008 [1.000] (0.948)	
*PAI-1- SNP*						
rs2227631	−0.176~0.15 (0.887)		−0.507~0.435 (0.913)		0.431~1.118 [0.694] (0.133)	
rs1799889 (4 G/4 G)	−0.28~0.025 (0.1)		0.012~0.888 (0.044*)	0.046~0.896 [0.471] (0.03*)	0.746~1.636 [1.104] (0.62)	
*PAI-1-associated SNPs*						
rs6976053	−0.146~0.129 (0.903)		−0.46~0.388 (0.868)		0.671~1.464 [0.991] (0.964)	
rs6486112	−0.18~0.133 (0.769)		−0.713~0.263 (0.365)		0.597~1.557 [0.964] (0.88)	
rs11128603	−0.271~0.501 (0.563)		−1.168~1.157 (0.922)		0.439~0.882 [1.967] (0.377)	
*IFNL3 SNP rs12979860 (CC)*	−01.371 ~4.7 (0.278)		−1.34~0.347 (0.248)		1.289~3.921 [2.248] (0.004*)	1.324~4.707 [2.496] (0.005*)

CI: confidence interval; OR: odds ratio; *p < 0.05; NA, not accessible; HCV: hepatitis C virus; PAI-1: plasminogen activator inhibitor-1; SVR: sustained virological response; BMI: body mass index; Log: logarithmic; HOMA-IR: homeostasis model assessment-estimated insulin resistance; ALT: alanine aminotransferase; APRI: aspartate aminotransferase to platelet ratio index; hsCRP: high sensitivity C-reactive protein; WBC: white blood cells; TC: total cholesterol; TGs: triglycerides; PAI-1: plasminogen activator inhibitor-1; BNP: brain natriuretic peptide; eGFR: estimated glomerular filtration rate; IFNL3: interferon-λ3.

**Table 3:** GEE analysis results of the 536 chronic hepatitis C patients who underwent anti-hepatitis C virus therapy.

Variants	PAI-1 (ng/ml)	
	95% CI of estimated exp (*β*) [Estimated exp. (*β*)]	*p*-values
Sex (female)	0.214~0.848 (0.426)	0.015*
Age	0.939~1.012 (0.975)	0.181
BMI	1.043~1.296 (1.163)	0.007*
Therapy intervention (without)	0.012~0.155 (0.043)	<0.001*
Therapy duration	0.841~1.346 (1.064)	0.553
SVR	0.202~1.514 (0.552)	0.249
HCV genotype	0.59~2.354 (1.178)	0.642
HCV RNA (Log10 IU/ml)	0.683~1.281 (0.935)	0.676
HOMA-IR	1.003~1.066 (1.034)	0.003*
ALT (U/L)	0.994~1.004 (0.999)	0.704
APRI	1.038~1.1 (1.069)	<0.001*
hsCRP (mg/dL)	0.941~1.279 (1.097)	0.235
WBC count (10^3^/μL)	0.982~1.253 (1.235)	0.07
Platelets count (10^3^/μL)	1.018~1.033 (1.026)	<0.001*
Hepatic steatosis (yes)	0.569~2.014 (1.07)	0.833
Liver cirrhosis (yes)	0.596~3.476 (1.495)	0.391
TC (mg/dL)	0.972~0.998 (0.985)	0.023*
TGs (mg/dL)	1.002~1.006 (1.004)	0.001*
PAI-1 (ng/ml)	NA	NA
BNP (Log_10_ pg/mL)	0.189~2.627 (0.704)	0.602
Homocysteine (*μ*mol/L)	0.234~3.86 (1.234)	0.885
eGFR	1.001~1.045 (1.023)	0.042*
PAI-1-rs1799889 (4 G/4 G genotype)	1.69~4.09 (2.63)	<0.001*
IFNL3-rs12979860 (CC genotype)	1.18~4.18 (2.22)	0.013*

Estimates of exp. (*β*), 95% confidence interval (CI) of exp. (*β*) and *p*-values for the variants that predicted PAI-1 longitudinally. GEE: generalized estimating equation; CI: confidence interval; OR: odds ratio; *p < 0.05; NA, not accessible; SVR: sustained virological response; BMI: body mass index; Log: logarithmic; HOMA-IR: homeostasis model assessment-estimated insulin resistance; ALT: alanine aminotransferase; APRI: aspartate aminotransferase to platelet ratio index; hsCRP: high sensitivity C-reactive protein; WBC: white blood cells; TC: total cholesterol; TGs: triglycerides; PAI-1: plasminogen activator inhibitor-1; BNP: brain natriuretic peptide; eGFR: estimated glomerular filtration rate; IFNL3: interferon-λ3.

**Table 4:** Comparison of the pre- and 24-week post-therapy variables in 536 chronic hepatitis C patients who underwent anti-HCV therapy stratified by the therapeutic response.

Variants	SVR (+), n = 445			SVR (−), n = 91		
	Pre-therapy value	Post-therapy value	Paired *t*-test for comparing pre- and post-therapy values *p-*values in patients with SVR	Pre-therapy value	Post-therapy value	Paired *t*-test for comparing pre- and post-therapy values *p-*values in patients without SVR
BMI	24.79+/−3.68	24.35+/−3.51	<0.001*	25.84+/−4.28	24.87+/−5.99	<0.001*
HCV RNA (Log_10_ IU/ml)	2.95+/−6.26	0.00+/−0.00	<0.001*	5.68+/−4.96	5.89+/−2.88	0.3222
HOMA-IR	3.02+/−6.56	2.83+/−3.96	0.5493	5.08+/−8.47	5.40+/−11.55	0.7332
ALT (U/L)	104.7+/−96.0	20.0+/−10.5	<0.001*	85.6+/−80.0	63.7+/−43.3	0.151
APRI	1.479+/−1.92	0.418+/−0.297	<0.001*	1.758+/−1.986	1.28+/−0.929	0.162
hsCRP (mg/dL)	1.70+/−3.16	1.86+/−4.54	0.607	1.59+/−1.97	1.91+/−3.6	0.436
WBC count (10^3^/μL)	5.83+/−1.92	5.79+/−1.77	0.689	5.59+/−1.66	5.16+/−1.22	0.628
Platelets count (10^3^/μL)	182.2+/−58.5	183.9+/−56.3	0.349	1.55.6+/−57.7	148.7+/−53.8	0.137
TC (mg/dL)	168.05+/−32.08	184.28+/−37.39	<0.001*	176.29+/−27.90	174.29+/−36.12	0.7021
TGs (mg/dL)	98.53+/−44.58	114.53+/−67.52	<0.001*	116.71+/−70.32	106.45+/−40.9	0.038*
PAI-1 (ng/ml)	6.93+/−3.47	9.08+/−4.43	0.003*	6.30+/−4.48	6.45+/−4.42	0.9355
BNP (pg/mL)	420.5+/−71.7	757.3+/−293.1	0.012*	512.0+/−47.1	499.6+/−52.6	0.869
Homocysteine (*μ*mol/L)	14.31+/−7.90	15.34+/−7.33	0.004*	13.76+/−4.72	13.77+/−4.52	0.8
eGFR	82.75+/−34.89	80.71+/−32.42	0.062	84.39+/−35.82	82.11+/−34.49	0.919

SVR: sustained virological response; HCV: hepatitis C virus; SVR: sustained virological response; BMI: body mass index; Log: logarithmic; *p < 0.05; HOMA-IR: homeostasis model assessment-estimated insulin resistance; ALT: alanine aminotransferase; APRI: aspartate aminotransferase to platelet ratio index; hsCRP: high sensitivity C-reactive protein; WBC: white blood cells; TC: total cholesterol; TGs: triglycerides; PAI-1: plasminogen activator inhibitor-1; BNP: brain natriuretic peptide; eGFR: estimated glomerular filtration rate.
