# Supplementary material for: Link between plasminogen activator inhibitor-1 and cardiovascular risk in chronic hepatitis C after viral clearance
Source: Sci Rep. 2017 Feb 13;7:42503. doi: 10.1038/srep42503 (PMC5304196; doi:10.1038/srep42503)
Supplement: Supplementary Tables [file srep42503-s1.doc]

**Link between plasminogen activator inhibitor-1 and cardiovascular risk in chronic hepatitis C after viral clearance**

**Ming-Ling Chang, Yu-sheng Lin**, **Li-Heng Pao, Hsin-Chih Huang, Cheng-Tang Chiu**

**Supplementary Table 1.** Univariate and multivariate analyses of factors associated with post-therapy PAI-1 levels in chronic hepatitis C patients with SVR.

|  | Post-therapy PAI-1 (ng/ml) | |
| --- | --- | --- |
| Post-therapy variants | Univariate analysis: 95% CI of estimated beta  (*p* values) | Multivariate analysis: 95% CI of estimated beta [estimated beta]  (*p* values) |
| Sex  (Male) | 0.213~2.051  (0.016*) | 0.086~1.782  [0.934]  (0.031*) |
| Age | -0.144 ~-0.066  (<0.001*) | -0.115~-0.033  [-0.074]  (0.001*) |
| BMI | 0.106~0.345  (<0.001*) | -0.012~0.257  [0.122]  (0.075) |
| ALT (U/L) | -0.11~0.055  (0.184) |  |
| APRI | -4.426~-1.499  (<0.001*) | -0.452~0.93  [0.239]  (0.496) |
| hsCRP | -0.083~0.102  (0.836) |  |
| WBC count | -0.036~1.779  (0.059) |  |
| Platelet count | 0.027~0.042  (<0.001*) | 0.016~0.037  [0.026]  (<0.001*) |
| TC (mg/dL) | -0.021~0.005  (0.224) |  |
| TG (mg/dL) | 0.001~0.24  (<0.001*) | 0.003~0.014  [0.006]  (0.206) |
| HOMA-IR | 0.005~0.291  (0.042*) | 0.092~0.336  [0.214]  (0.01*) |
| Hepatic steatosis | 0.229~2.070  (0.015*) | -0.635~1.152  [0.259]  (0.569) |
| Liver cirrhosis | -3.966~-1.789  (<0.001*) | -1.599~0.792  [-0.403]  (0.507) |
| Log BNP | -0.856~1.970  (0.438) |  |
| Homocysteine | -0.145 ~0.39  (0.257) |  |
| eGFR | -0.022~0.009  (0.396) |  |
| *PAI-1- SNP*  rs2227631  rs1799889  (4G/4G)  *PAI-1-associated SNP*  rs6976053  rs6486112  rs11128603 | -0.425~0.997  (0.428)  0.045~1.291  (0.036*)  -0.254~0.95  (0.255)  -0.956~0.466  (0.497)  -2.694~0.699  (0.238) | -0.886~0.231  [-0.328] (0.249) |
| IFNL3 SNP rs12979860 (CC) | -0.391~2.581  (0.148) |  |

CI: confidence interval; OR: odds ratio. *: *p*<0.05; NA, not accessible; HCV: hepatitis C virus; PAI-1: plasminogen activator inhibitor-1; SVR: sustained virological response; BMI: body mass index; Log: logarithmic; HOMA-IR: homeostasis model assessment-estimated insulin resistance; ALT: alanine aminotransferase; APRI: aspartate aminotransferase to platelet ratio index; hsCRP: high sensitivity C- reactive protein; WBC: white blood cells; TC: total cholesterol; TGs: triglycerides; PAI-1: plasminogen activator inhibitor-1; BNP: brain natriuretic peptide; eGFR:estimated glomerular filtration rate; SNP: single nucleotide polymorphism; IFNL3: interferon-λ3

**Supplementary Table** 2. Prevalence of conventional cardiovascular risks of the CHC patients with and without SVR.

| Risk factors | SVR patients (n=455) | Non-SVR patient  (n=91) | P values |
| --- | --- | --- | --- |
| Sex, male, n (%) | 257 (57.8) | 47 (52) | 0.394 |
| Age | 53.04+/-12.93 | 57.5+/-12.47 | 0.160 |
| BMI | 24.79+/-3.68 | 25.84+/-4.28 | 0.057 |
| Smoking, n (%) | 114 (25.1) | 25 (27.5) | 0.965 |
| Diabetes history, n (%) | 46 (10.1) | 16 (17.6) | 0.131 |
| Hypertension, n (%) | 193 (42.4) | 40 (43.9) | 0.847 |
| Dyslipidemia, n (%) | 82 (18.0) | 16 (17.6) | 0.829 |

CHC: chronic hepatitis C; SVR: sustained virological response; BMI: body mass index

**Supplementary Table 3. List of SNPs of PAI-1 analyzed and the ID numbers of TaqMan assays**

| SNP | ID number of TaqMan assays |
| --- | --- |
| rs2227631 | C_2620923_10 |
| rs6976053 | C_32067460_10 |
| rs6486122 | C_2160490_10 |
| rs11128603 | AHZAG02 |
| rs1799889 |  |

SNP: single nucleotide polymorphism; PAI-1: plasminogen activator inhibitor-1
